# The First Advent of the Whole Genome Sequencing Cohort in Japan

**DOI:** 10.31662/jmaj.2025-0389

**Published:** 2025-09-26

**Authors:** Yutaka Suzuki

**Affiliations:** 1 Life Science Data Research Center, The University of Tokyo, Tokyo, Japan

**Keywords:** whole genome sequencing, reference Japanese genomes, cohort study, omics analysis, analytical pipeline

The first decade was over in the blink of an eye. Since its launch in 2013, the Tohoku Medical Megabank Organization (ToMMo) has generated a cohort of 150,000 healthy Japanese individuals. This cohort consists of two parts (see Figure 1 in the Katsuoka et al. ^(1)^). The first part is the ‘Community-based cohort,’ comprising 80,000 randomly selected individuals. The second is the ‘Three-generation cohort,’ comprising 16,757 pairs of parents, 9,038 trios, and 181 families, starting from grandparents. Whole genome sequencing analysis has been started for these individuals, and by gradually increasing the number, 100,000 individuals have now been sequenced (Figure 3 in the Katsuoka et al. ^(1)^). Even after the whole genome sequencing analysis, these individuals are followed to collect a wide variety of health- and lifestyle-related data, such as blood test results, oral health data, and responses to detailed questionnaires. Now the obtained sequence data are being used as standard genomes for the Japanese population, providing a reference control for various research purposes, such as identifying disease genes from a medical viewpoint, and conducting epidemiological analyses of the Japanese population from a basic scientific viewpoint. The success of the ToMMo project has opened up new fields for various types of genome study.

In this volume of *JMA*, Katsuoka et al. ^(1)^ look back at the history of ToMMo. They describe the detailed protocols for their in-house operations and quality control ^(1)^. These precious pieces of information record the attempts to launch the first Japanese genome community and leave a substantial legacy for future generations. When ToMMo was first launched, the procedure for large-scale data production sequencing was not established at all. Furthermore, ToMMo itself had to be established for this purpose, where the first task was, therefore, to design the experimental laboratory and computational facilities, as well as creating thousands of start-up jobs and providing initial training for technical staff. The founding group of designated researchers may have been overwhelmed by the difficulties they faced. Nevertheless, they joined forces to overcome these difficulties one-by-one, embodying the spirit of their team name, “ToMMo,” which means “friendship” in Japanese.

Even in later years, alongside the updating of the sequencers, the procedures for library preparation and data analysis, accompanied by careful quality control at each stage, have changed (Figure 3 in the Katsuoka et al. ^(1)^). These changes include a major update of the sequencers, from Illumina’s HiSeq2500 to NovaSeq and BGI’s DNBSEQ. Sometimes drastic changes to both the hardware and software must be reflected in the analytical pipeline without delay, as it is in full operation and cannot be stopped long term. In this paper, Katsuoka et al. ^(1)^ describe the details of the optimizations, ranging from library concentration, inset length and laboratory automation. Although this knowledge is now outdated, there are many lessons to be learned for future schemes.

The challenge they encountered was not only scientific, but also other social or political issues. Sometimes, inherent to the nature of a health cohort study of this kind, the obtained scientific results were not always highly regarded. Although many types of medical research would not be possible without a solid foundation of the normal controls, the disease studies themselves were occasionally the ones which were given the highest reputation. Although the health and medical information collected on each individual could potentially be a powerful resource, its power would not be revealed until the clinical symptoms of each individual become relevant. There was a time when substantial criticism was leveled, calling for a significant reduction in the budget, which would make it impossible to maintain the samples that had already been collected. They had to wait a long time for the results of their work to become apparent.

Eventually, the time has proven that the ToMMo’s approach was in the right direction. Nowadays, large-scale cohorts are becoming a national asset, especially in developed countries. In the United Kingdom (UK), Genomics England was launched, based on the National Health Service, where genomic sequence data and related personal health information are collected on a large scale ^(2)^. By 2025, the whole genome sequencing of half a million individuals will have been completed, and this number is expected to increase tenfold within the next decade. Similar initiatives have been launched in the United States and other countries worldwide, including China and Singapore. The consensus is that, in the long run, such cohort studies are worth a large investment. As commercial applications increase, large-scale analysis, especially deoxyribonucleic acid (DNA) sequencing, is gradually shifting from academic laboratories to private service provider companies. Over the next decade, ToMMo will face qualitatively different challenges, although the underlying spirit will remain the same.

The next decade will be an even more challenging one. The first advent of large-scale cohort studies has made it gradually clear that the current approach relying on the genomic DNA sequencing has its limits. Even with the help of the latest artificial intelligence technologies, it may be difficult to extract all the necessary biological information from the DNA code, At least, interactions with environmental factors are not encoded there. In our bodies, trillions of cells read the DNA sequence to produce messenger ribonucleic acid, proteins, metabolites, and molecules from other omics layers. Direct measurements of these molecules could help to elucidate the biological meaning of the genomic DNA code. While it may not be possible to analyze all of these cells, new technologies are being developed to enable high-resolution assays that were unimaginable 10 years ago. For example, current single-cell and spatial analysis can analyze gene expression profiles at single-cell resolution for millions of cells, and the latest protein analysis can measure up to 10,000 proteins from a tiny serum sample. Initial trials to adopt these technologies in several cohort studies have already begun, including the UK Biobank studies ^(3), (4)^. As described by Katsuoka et al. ^(5)^, “Telomere-to-Telomere” grade genome sequencing is also starting. Indeed, this is another transformative era for genome sciences. Let us see how ToMMo’s next leap will bring about the new genomic era within the next decade.

## Article Information

### Conflicts of Interest

None
